# Assessment of Creativity Potential of a 3DGAN in Implant Crown Design: A Proof-of-Concept Study

**DOI:** 10.3390/jfb17070324

**Published:** 2026-07-05

**Authors:** Aleksandar Naydenov, Todor Uzunov, Dimitar Kirov, Georgi Kostadinov

**Affiliations:** 1 Faculty of Dental Medicine, Medical University of Sofia, 1431 Sofia, Bulgaria; uzunov@fdm.mu-sofia.bg (T.U.); d.kirov@fdm.mu-sofia.bg (D.K.); 2 Department of Information Technologies, New Bulgarian University, 1618 Sofia, Bulgaria; grgkostadinov@gmail.com

**Keywords:** artificial intelligence, 3DGAN, creativity, generalization, implants, design

## Abstract

Digital dentistry increasingly relies on artificial intelligence (AI) to automate restorative design. However, the ability of generative networks to produce multiple geometrically distinct outputs for the same prosthetic field remains insufficiently evaluated. This study assessed repeated-output geometric variability in a previously developed three-dimensional generative adversarial network (3DGAN) for screw-retained implant crown design as a preliminary indicator of potential generative diversity. Nine AI-generated implant crown designs were analyzed, consisting of three independently generated crowns for each of three different prosthetic fields. Within each set, the crowns were superimposed and compared using “MeshLab”. Mean Hausdorff distance (HD), maximum HD, and root mean square (RMS) values were recorded, with 0.05 model units used as the threshold for identifying insufficient morphological variation. The overall mean HD was 3.32 model units, the mean maximum HD was 16.18 model units, and the mean RMS value was 4.40 model units. No pairwise comparison showed values equal to or below 0.05 model units. In conclusion, the investigated 3DGAN demonstrated preliminary evidence of geometric output variability compatible with potential generative diversity.

## 1. Introduction

The digital transformation of prosthetic dentistry has created favorable conditions for the integration of artificial intelligence (AI) into restorative workflows [1,2]. Computer-aided design (CAD) and manufacturing systems have improved precision and reproducibility in implant-supported restorations, yet final crown morphology remains strongly dependent on CAD operators [1,2]. This dependence is clinically important because crown design must reproduce individualized occlusal morphology, proximal contacts, axial contour, and integration with the antagonist and adjacent dentition [3]. In implant-supported crowns, the design task is even more specific because the restoration must also respect the emergence profile, implant position, and screw-access channel, while maintaining functional occlusal contacts [4].

Generative neural networks offer a different approach by learning morphological patterns directly from data and then producing restorations fully automatically [5,6,7,8]. Recent studies have shown that deep learning and generative adversarial network (GAN) based software can reduce crown-design time and can produce restorations with clinically relevant morphology, occlusion, internal fit, and proximal contacts [3,9]. In addition, recent implant-specific evidence indicates that deep learning-designed implant-supported posterior crowns can be evaluated not only by design time, but also by tooth morphology, emergence profile, occlusion, and proximal contacts [4].

Within this context, evaluating an AI system only by speed or basic geometric similarity is insufficient. For clinical relevance, such a system should also be able to generate more than one design solution for a given case and remain functional when the prosthetic field differs from the exact configurations encountered during training process of the network [10,11,12,13]. This is especially relevant for generative models, because realistic output and output diversity represent complementary properties: a model should generate anatomically plausible crowns, but it should not simply repeat the same solution for the same input [14]. Three-dimensional dental data also requires specific evaluation, because the generated crown is not a flat image but a complex surface with functional anatomical details [15].

The present article focuses on investigation of the creative potential of a previously developed three-dimensional generative adversarial network (3DGAN) for screw-retained implant crowns [13]. Creative potential was interpreted as the ability to produce multiple morphologically distinct designs as an output from the same input field while preserving plausibility [11,12,13]. In the context of AI-based implant crown design, creative potential also may be defined as the capacity of a generative model to produce outputs that are not identical repetitions of the training data or of each other, but are instead novel, task-appropriate, and clinically valuable morphological alternatives for the same prosthetic situation [16]. The aim of the study was therefore to assess whether the investigated 3DGAN can generate multiple individualized crown designs.

## 2. Materials and Methods

**Figure 1:**
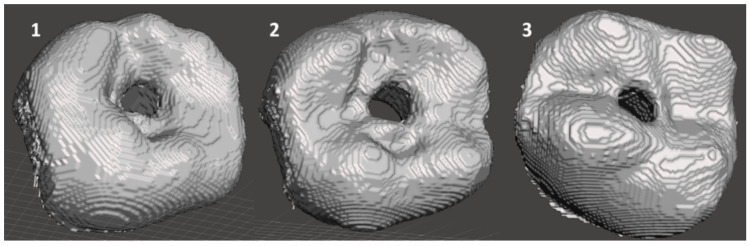
The three newly generated crowns produced by the three-dimensional generative adversarial network (3DGAN), used as Set No. 1 for investigating the creative potential of the network. Each crown—1, 2, and 3—was superimposed against the others within the set in MeshLab 2025.07 to measure the Hausdorff distance (HD).

**Figure 2:**
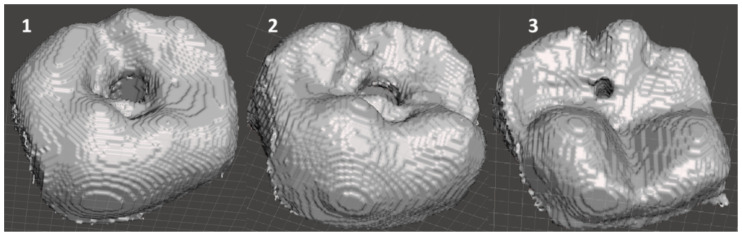
The three newly generated crowns produced by the 3DGAN, used as Set No. 2 for analyzing the creative potential of the network. Each crown—1, 2, and 3—was superimposed against the others within the set in MeshLab 2025.07 to measure the HD.

**Figure 3:**
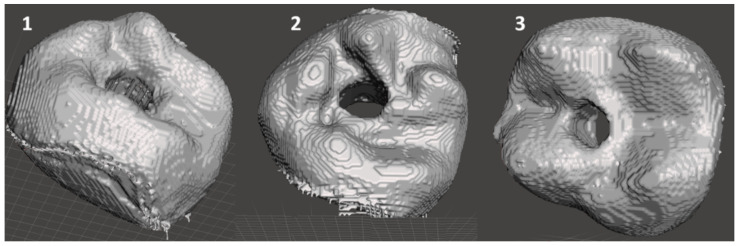
The three newly generated crowns produced by the 3DGAN, used as Set No. 3 for analyzing the creative potential of the network. Each crown—1, 2, and 3—was superimposed against the others within the set in MeshLab 2025.07 to measure the HD.

Nine screw-retained implant crown designs were using a custom-developed three-dimensional generative adversarial network (3DGAN) created by G. Kostadinov and A. Naydenov [13] and then analyzed. For crown generation, three different prosthetic fields were selected. These prosthetic fields were part of the validation set used to evaluate the 3DGAN and had not been seen by the network during training [13]. For each prosthetic field, three independent crown designs were generated by the network. Three generated crowns per field were selected because this number allows pairwise comparison within each field: crown 1 versus crown 2, crown 1 versus crown 3, and crown 2 versus crown 3. Thus, the study sample consisted of nine generated implant crown designs and nine pairwise geometric comparisons. The generated designs were numbered and are presented in Figure 1, Figure 2 and Figure 3. MeshLab, version 2025.07 (Visual Computing Lab, Institute of Information Science and Technologies, National Research Council of Italy [ISTI-CNR], Pisa, Italy), was used to compare the generated crowns within each set by superimposition and Hausdorff distance (HD) analysis.

Creative potential was considered present when, within each of the three investigated sets, the recorded HD parameters—maximum HD, mean HD, and root mean square (RMS)—showed values greater than the predefined tolerance threshold of 0.05 model units. Conversely, lack of creative potential was defined as the presence of at least one pairwise comparison with HD values equal to 0.00 or lower than 0.05 model units. The standard tessellation language (STL) models were exported in millimeters from DentalCAD, version DentalCAD 3.2 Elefsina (exocad GmbH, Darmstadt, Germany), and were imported into MeshLab without normalization, scaling, or other dimensional transformation. Therefore, one coordinate unit in MeshLab corresponded to 1 mm in the original dental model, and the predefined threshold of 0.05 model units was equivalent to 0.05 mm, or 50 µm.

## 3. Results

**Figure 4:**
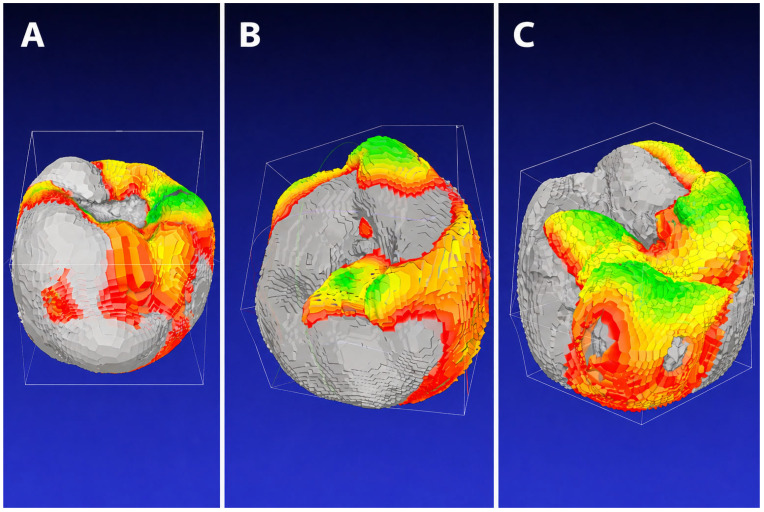
Visualization of the superimposed pairs of standard tessellation language (STL) files (1, 2, and 3) generated by the 3DGAN using the prosthetic field from STL file No. 1 as input. The histogram is visible on the left side of each of the three images, with the individual colors representing the discrepancies between the files. (**A**)—Crown 1 superimposed on Crown 2. (**B**)—Crown 1 superimposed on Crown 3. (**C**)—Crown 2 superimposed on Crown 3.

**Figure 5:**
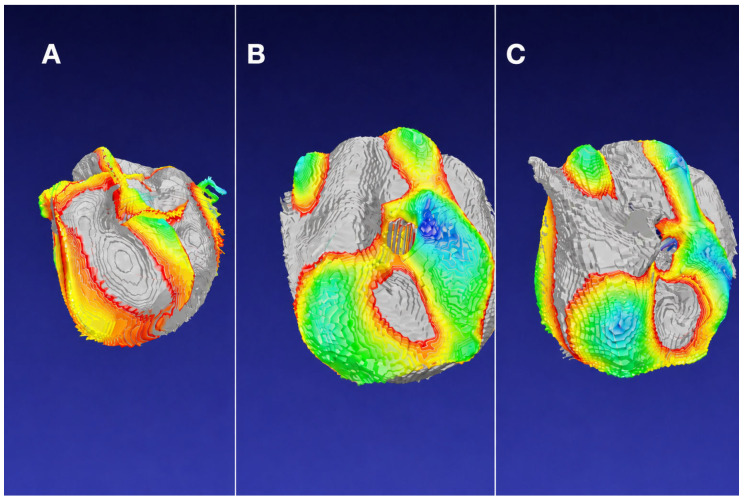
Visualization of the superimposed pairs of STL files (1, 2, and 3) generated by the 3DGAN network using the prosthetic field from STL file No. 2 as input. The histogram is visible on the left side of each of the three images, with the individual colors representing the discrepancies between the files. (**A**)—Crown 1 superimposed on Crown 2. (**B**)—Crown 1 superimposed on Crown 3. (**C**)—Crown 2 superimposed on Crown 3.

**Figure 6:**
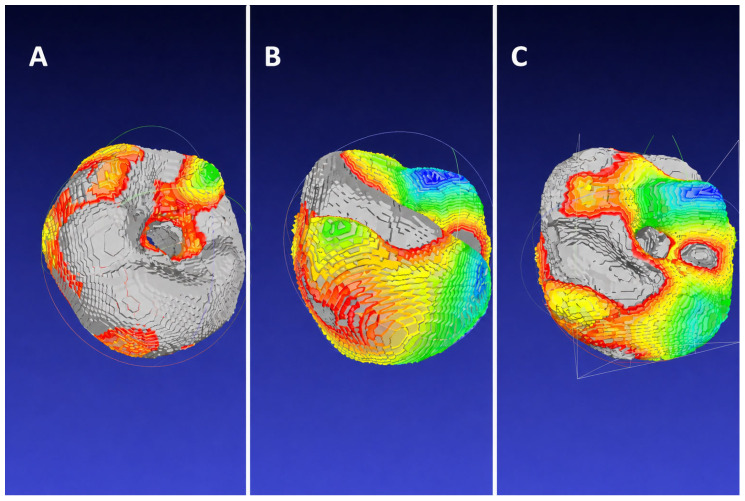
Visualization of the superimposed pairs of STL files (1, 2, and 3) generated by the 3DGAN network using the prosthetic field from STL file No. 3 as input. The histogram is visible on the left side of each of the three images, with the individual colors representing the discrepancies between the files. (**A**)—Crown 1 superimposed on Crown 2. (**B**)—Crown 1 superimposed on Crown 3. (**C**)—Crown 2 superimposed on Crown 3.

Table 1 presents the values recorded in model units for the Hausdorff distance between the compared pairs after superimposition in MeshLab 2025.07. Figure 4 shows the examined Set No. 1, which includes three automated wax-ups on the same prosthetic field; Figure 5 shows the examined Set No. 2, which includes three automated wax-ups on the same prosthetic field; and Figure 6 shows the examined Set No. 3, which includes three automated wax-ups on the same prosthetic field.

Results for HD: The mean HD value is 3.32. The lowest value is 1.08, observed in the studied pair 1 → 2, generated relative to STL file No. 1. The highest value is 5.14, observed in the studied pair 2 → 3, generated relative to STL file No. 3.

Results for “max HD”: The mean value is 16.18. The lowest value is 12.20, observed in the studied pair 1 → 2, generated relative to STL file No. 1. The highest value is 23.32, observed in the studied pair 2 → 3, generated relative to STL file No. 3.

Results for RMS: The mean value is 4.40. The lowest value is 1.84, observed in the studied pair 1 → 2, generated relative to STL file No. 1. The highest value is 6.74, observed in the studied pair 2 → 3, generated relative to STL file No. 3.

No values less than or equal to 0.05 model units were recorded for any of the Hausdorff distance parameters investigated (Table 1).

## 4. Discussion

The present results should be interpreted within the broader context of generative artificial intelligence in dental crown design. Recent evidence shows that AI-based crown design is no longer only a theoretical concept, but an emerging restorative workflow with measurable performance in morphology, accuracy, fit, and time efficiency. Ding et al. used a true three-dimensional deep convolutional generative adversarial network for automatic crown design and reported that the generated crowns showed the lowest morphological discrepancy compared with other design methods, as well as biomechanical behavior close to that of natural teeth. However, these findings were obtained specifically for lithium disilicate restorations in the premolar region and should not be directly generalized to other restorative materials or tooth regions without further validation [17]. Similarly, Liu et al. demonstrated that AI-designed restorations can improve efficiency and accuracy, reporting reduced design time and clinically relevant reproducibility compared with conventional workflows [18]. These findings support the relevance of evaluating not only whether an AI system can generate a crown, but also whether it can generate morphologically plausible and usable crown designs.

Kong et al. reported that AI applications in crown prostheses include crown design, crown evaluation, finish-line detection, preparation assessment, and prognosis prediction, while also noting that many studies still use small datasets and do not fully disclose the underlying algorithms [19]. A later scoping review focused specifically on AI-designed crowns found that most studies evaluated morphological accuracy, design time, and occlusal contacts, but also concluded that standardized evaluation protocols and further clinical validation are still needed [20]. This supports the methodological importance of the present study, because the use of repeated generation on the same prosthetic field and quantitative superimposition provides direct information about output variability.

For a long time, creativity was not regarded as a core property of artificial intelligence systems. The working definition of computational creativity is the creation of something new and valuable, often also “surprising” [16].

The indicators selected in the present study for investigating creativity/diversity in generative models—precision and recall—distinguish quality (precision) from coverage/diversity (recall) and represent a standard for evaluating generative models beyond one-dimensional metrics [21]. Other commonly used values include Fréchet Inception Distance (FID), which correlates with perceived quality and was introduced by Heusel et al. [22]. For three-dimensional shapes, coverage, minimum matching distance (MMD), and Jensen–Shannon divergence (JSD) represent de facto metrics for diversity and similarity, as demonstrated by Achlioptas et al. [23].

Another relevant question concerns the factors that influence creativity in GAN-based systems. We found that the training data used and the network architecture provide controllable variability without loss of plausibility [24]. BigGAN networks also exist, which improve latent noise during generation [25].

The creative potential in the present study was interpreted as controlled morphological variation: the ability to generate different crown designs for the same input field, while maintaining geometrically plausible morphology. This interpretation is consistent with recent work on generative model evaluation, which emphasizes that fidelity and diversity are complementary aspects of performance and that common metrics may be insufficient when used alone [26].

The relevance of diversity assessment is even greater for three-dimensional data. The recent 3D-generation literature emphasizes that the evaluation of generated shapes should include not only realism or surface similarity, but also diversity, coverage, and geometric consistency [27].

The results obtained from the superimposition of three independent automated digital designs on the same prosthetic field, across three sets, show that the network generates different yet plausible solutions: mean Hausdorff distance (HD) of approximately 3.32 model units, maximum HD of approximately 16.18 model units, and RMS of approximately 4.40 model units. There were no superimposed pairs with HD ≤ 0.05; in other words, the network does not replicate the same solution and demonstrates characteristics of creative potential. This is desirable behavior in tasks involving multiple geometrically acceptable morphologies, as it allows selection of the “most suitable” crown according to specific priorities, such as contacts, screw-access channel, and fissure aesthetics.

The threshold of 0.05 model units was adopted as a predefined analytical tolerance for distinguishing near-coincident from non-coincident mesh outputs, rather than as a universal clinical cutoff. This threshold was used in different 3D-comparison studies demonstrating that Hausdorff mean, maximum, and RMS measurements can quantify subtle surface discrepancies in the hundredths-of-a-unit range, as well as by deviation-analysis protocols using ±0.05 mm as the tolerance interval for local surface correspondence [28,29]. Accordingly, values exceeding 0.05 model units were interpreted in the present proof-of-concept study as indicating that the generated meshes were not geometrically identical within the selected analytical tolerance. As all recorded HD parameters were substantially greater than 0.05 model units, the conclusion of morphological non-identity was not dependent on borderline measurements around the selected threshold.

The preprocessing pipeline, detailed 3DGAN architecture, training dataset size, case-selection criteria, and training parameters were previously described in detail by Kostadinov and Naydenov [13].

Several limitations of the present study should be discussed. The investigation was performed on a limited dataset, including only three prosthetic fields and nine generated implant crown designs. Therefore, the results should be interpreted as preliminary evidence of measurable geometric output variability rather than clinical plausibility, or clinical performance of the 3DGAN. In addition, the present investigation was designed as a proof-of-concept observational study of repeated 3DGAN outputs rather than as a comparative study between independent experimental groups; no inferential statistical analysis was performed. The results were analyzed descriptively using geometric comparison parameters, including mean Hausdorff distance, maximum Hausdorff distance, and RMS values for each pairwise comparison. The present investigation did not evaluate or validate clinical plausibility, prosthodontic function, occlusal adequacy, emergence profile, marginal adaptation, biomechanical performance, or clinical usability. The Hausdorff-distance values obtained in MeshLab may be influenced by mesh resolution, vertex distribution, surface sampling, registration, and outlier handling, and the absolute values may differ when metrology-grade software such as Geomagic Control X or ZEISS INSPECT is used.

An additional limitation is that the generated crowns were evaluated only as digital meshes and were not validated with respect to restorative materials or manufacturing workflows, including milling or 3D printing. Furthermore, because the generated meshes smoothing was performed using Blender [13], the independent contribution of the 3DGAN to the final surface quality and manufacturability could not be exactly determined.

Despite these limitations, the study has clinical relevance because it demonstrates that the investigated 3DGAN can generate multiple morphologically different implant crown designs from the same prosthetic field. This is important because, in clinical practice, more than one crown morphology may be acceptable for the same implant situation, and the final choice may depend on occlusion, esthetics, screw-access position, hygiene, and clinician preference. Therefore, such AI-based generation could support digital wax-up selection and reduce dependence on manual CAD design.

## 5. Conclusions

The recorded mean values indicate that the assessed 3DGAN possesses measurable output variability compatible with potential generative diversity. These preliminary findings show that the network can generate multiple geometrically non-identical implant crown designs from the same prosthetic field.

## Figures and Tables

**Table 1:** Hausdorff distance (HD) and root mean square (RMS) in each compared pair of designs.

Prosthetic Field (Input Information)	Compared Pair of Designs	HD Results		
		Mean HD	Max. HD	RMS
No. 1	1 vs. 2	1.12	12.20	1.84
	1 vs. 3	3.87	17.26	4.96
	2 vs. 3	4.31	16.20	5.35
No. 2	1 vs. 2	1.32	16.40	2.42
	1 vs. 3	4.00	14.98	5.01
	2 vs. 3	4.13	14.35	5.16
No. 3	1 vs. 2	1.08	12.40	1.87
	1 vs. 3	4.89	18.49	6.26
	2 vs. 3	5.14	23.32	6.74

## Data Availability

The datasets used and analyzed during the current study are available from the corresponding author on reasonable request.

## Abbreviations

The following abbreviations are used in this manuscript:

AI	Artificial Intelligence
CAD	Computer-aided design
FID	Fréchet Inception Distance
GAN	Generative Adversarial Network
HD	Hausdorff Distance
JSD	Jensen–Shannon divergence
MMD	Minimum matching distance
RMS	Root Mean Square
STL	Standard Tessellation Language
3DGAN	Three-dimensional generative adversarial network
